# Effects of Pelvic Stabilization Training with Lateral and Posterior Tilt Taping on Pelvic Inclination, Muscle Strength, and Gait Function in Patients with Stroke: A Randomized Controlled Study

**DOI:** 10.1155/2022/9224668

**Published:** 2022-09-05

**Authors:** Kyoung-Sim Jung, Jin-Hwa Jung, Tae-Sung In, Hwi-Young Cho

**Affiliations:** ^1^Department of Horse Industry, Sungwoon University, Yeongcheon, Republic of Korea; ^2^Department of Occupational Therapy, Semyung University, Jecheon, Republic of Korea; ^3^Department of Physical Therapy, Gimcheon University, Gimcheon, Republic of Korea; ^4^Department of Physical Therapy, Gachon University, Incheon, Republic of Korea

## Abstract

**Background:**

This study was aimed at investigating the effect of pelvic tilt taping on muscle strength, pelvic inclination, and gait function in patients with stroke.

**Methods:**

A total of 60 patients with stroke were included in our study and randomly divided into three groups: the posterior pelvic tilt taping (PPTT, *n* = 20), the lateral pelvic tilt taping (LPPP) with PPTT (LPPP+PPTT, *n* = 20), and the control (*n* = 20) groups. All participants performed pelvic stabilization exercises consisting of 6 movements: supine, side lying, quadruped, sitting, squatting, and standing (30 min/day, five days/week, for six weeks). PPTT to correct anterior pelvic tilt was applied to the LPTT+PPTT and PPTT groups, and lateral pelvic tilt taping was additionally applied to the LPTT+PPTT group. LPTT was performed to correct the pelvis tilted to the affected side, and PPTT was performed to correct the anterior pelvic tilt. The control group did not undergo taping. A hand-held dynamometer was used to measure the hip abductor muscle strength. In addition, a palpation meter and 10-meter walk test were used to assess pelvic inclination and gait function.

**Results:**

Muscle strength was significantly stronger in the LPTT+PPTT group than in the other two groups (*p* = 0.01). The anterior pelvic tilt was significantly improved in the taping group compared to the control group (*p* < 0.001), and the lateral pelvic tilt was significantly improved in the LPTT+PPTT group compared to the other two groups (*p* < 0.001). Significantly greater improvements in gait speed were observed in the LPTT+PPTT group than in the other two groups (*p* = 0.02).

**Conclusions:**

PPPT can significantly affect pelvic alignment and walking speed in patients with stroke, and the additional application of LPTT can further strengthen these effects. Therefore, we suggest using taping as an auxiliary therapeutic-intervention method in postural control training.

## 1. Background

Pelvic stability is the ability to control muscle activity between the lower trunk and hip muscles to provide proximal dynamic stability and allow efficient lower-limb mobility during functional balance and mobility tasks [1]. However, patients with stroke suffer from spasticity, weakness, inappropriate muscle activity patterns, and changes in trunk and pelvic alignment due to neurological damage. These symptoms interfere with proper postural control performance [2]. Also, it was reported that patients with stroke had the pelvic tilted forward to the most affected side in the standing position. This pelvic asymmetry was also significantly correlated with weight-bearing asymmetry and was found to have a higher correlation with lateral tilt [3]. The gluteus medius muscle also acts as a stabilizer of the pelvis during weight bearing. However, in a study measuring the hip muscle activity during the application of perturbations in a standing position in patients with stroke, the activity of the most affected side abductor decreased, and the least side adductor was excessively used [4]. Pelvic control is crucial in humans' ability to efficiently and economically perform functional movements such as gait and balance [5]. However, if pelvic control is not properly induced during movement due to damage to the nervous system or musculoskeletal system, gait speed, stability, and efficiency may be reduced in patients, or secondary problems such as falls may occur [6–8].

Various therapeutic interventions such as cycling [9], treadmill [10], robot-assisted gait training [11], aquatic therapy [12], and taping [13] are being used in clinical practice to improve the walking function of stroke patients. Among these interventions, taping is easy to apply without side effects. It can also be used to control muscle tonus or correct joints depending on the application method [14]. Taping could promote muscle activity by activating proprioceptive receptors or increasing the excitability of motor nerves when applied to the skin [15–17]. It is also used to correct alignment by relaxing shortened muscles or strengthening weakened muscles in patients with stroke and musculoskeletal problems [18]. In a study confirming the immediate effect of taping and Thera Togs on the hip abductor, both reported that muscle activity and walking speed were increased in patients with stroke [19]. Mehta et al. reported that due to attaching tapings to the thoracic spine and abdominal muscles in patients with stroke, anterior pelvic tilt, and pelvic obliquity were significantly improved in a sitting position. They argued that more abdominal muscles were recruited as taping improved the active musculotendinous and neural control components of the spine stabilizing system [20]. Also, in a study in which taping was attached to the back muscle in children with cerebral palsy, the kyphosis angle and Gross Motor Function Measure Sitting score were significantly improved compared to the control group [21]. In a meta-analysis, taping has the effect of improving spasticity, motor function, and balance. Still, it is more effective to use it as an assistive device during exercise training than to use it alone [22].

Pelvic stabilization exercise (PSE) trains cocontraction and selective contraction of the lower trunk and proximal hip muscles in lying, sitting, and standing positions. It has been reported that PSE positively affects hip strength and walking speed in stroke patients [23]. However, no studies have analyzed the long-term effects of PSE combined with the pelvis supported by taping for postural correction in stroke patients. In addition, in a study combining functional exercise and pelvic posterior tilt taping, the anterior pelvic tilt, hip extensor strength, and walking speed were significantly improved in patients with stroke. They argued that the improvement of postural alignment through pelvic tilt taping during weight-bearing on the most affected side is due to appropriate stimulation to the hip extensors [24]. However, it was not confirmed whether there was a difference in the effect of applying lateral pelvic tilt taping (LPTT) and posterior pelvic tilt taping (PPTT) simultaneously and applying only PPTT. This study hypothesized that applying LPTT and PPTT with PSE would improve muscle strength, pelvic alignment, and gait ability compared to PPTT alone with PSE.

## 2. Material and Methods

### 2.1. Study Design and Setting

This study was designed as a single-blind randomized controlled trial and was conducted at a specialized rehabilitation center for inpatients with neurological disorders.

### 2.2. Participants

The study was conducted from March to June 2021, targeting stroke patients hospitalized at K Hospital in Gyeonggi-do. The participants were recruited with the recommendation of the therapist in charge. This study included hemiplegic patients who (1) could understand and follow verbal commands, (2) could walk 15 meters independently without assistance, (3) had no neglect and sensory loss, (4) had unilateral hemiplegia, and (5) had a stroke for the first time with a motor recovery of the lower extremity of Brunnstrom stage 3 or higher. In addition, we excluded those with (1) vestibular impairment, (2) neglect and sensory loss, (3) age less than 30 years and more than 65 years, (4) cardiopulmonary system problems, (5) skin wounds in the upper limb or lower limb, (6) musculoskeletal disorders, and (7) orthopedic diseases affecting gait [9].

### 2.3. Experimental Procedure

We calculated the sample size using G∗Power 3.1.9.2 software. In this pilot study (12 patients), the mean power, alpha error, and effect size were 0.8, 0.05, and 0.46, respectively. As a result of G∗power software analysis, the acceptable sample size was at least 51 patients. Therefore, this study included 63 patients considering drop-outs. Informed consent was obtained after explaining the study to all the patients. The study protocol was approved by the Gachon University Institutional Review Board (1044396-202104-HR-072-01) and was registered in the clinical trial registry (no. KCT0006261).

Sixty-three participants with chronic hemiparesis were included in this study and randomly distributed into three groups using a section envelope:

1. Pelvic stability exercise with taping applied to the lateral and posterior pelvic tilt (LPTT+PPTT group, 21 participants)
2. Pelvic stability exercise with taping applied to the posterior pelvic tilt (PPTT group, 21 participants)
3. Pelvic stability exercise only (control group, 21 participants)

**Figure 1:**
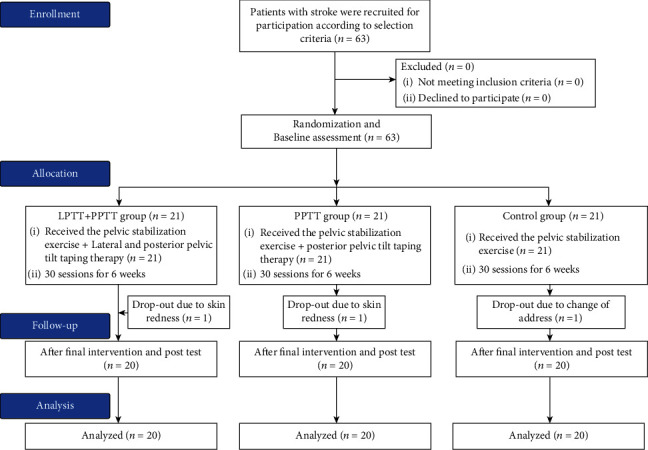
Flow diagram of participants through the study.

In the LPTT+PPTT group and the PPTT group, one person each dropped out due to skin redness due to taping, and in the control group, one person dropped out due to address change. A total of 60 people received a postevaluation (Figure 1).

### 2.4. Intervention

All three groups performed pelvic stability exercises for 30 minutes daily, five times a week for six weeks. The pelvic stability exercise was modified by referring to Dubey's method [23] and consisted of six movements in supine, side-lying, quadruped, sitting, squat, and standing postures to activate the lower trunk and proximal hip muscles:

1. The pelvic bridge was performed by lifting the pelvis off the floor in a hook-lying position. The difficulty of movement was gradually increased by executing it with one leg or on a balance ball
2. The participants were lying on the least affected side with the hip and knee joints flexed, and both knees were lifted toward the ceiling to perform pelvic rotation. The therapist increased the difficulty by providing resistance to hip abduction from the lateral side of the pelvis
3. The arms and legs were alternately raised in the quadrupedal position. The difficulty level was raised by lifting crossed arms and legs simultaneously or by applying a balance pad under the hand
4. In a sitting position, both arms were reached in the diagonal direction, or one leg was raised alternately. Balance pads were applied under the buttocks and the feet to gradually increase the intensity. The participants were also encouraged to keep their torso upright during the exercise
5. They were asked to maintain a squat position with their back against the wall. At this time, the pelvis is tilted posteriorly to attach the waist to the wall. Separating the pelvis, lower, and upper trunk from the wall segment by segment increases the intensity by maintaining the squat position without leaning on the wall
6. Weight shift training was performed through forward and diagonal reach-out in a standing position with one foot in front or on the stairs, and the difficulty was increased by raising the reaching distance or the height of the stairs

Each movement was maintained for 5 seconds, and three sets were performed ten times. In addition, they were allowed to rest for 1 minute between the sets.

All therapeutic training was performed by physical therapists with more than five years of clinical experience in rehabilitation exercises for stroke patients. The intervention was conducted in a space where a safety bar was installed within a distance of the patient's hand so that the subject could feel anxious or respond to emergencies during the intervention.In addition, the researchers educated the participants in advance to express to them immediately if they felt excessive fatigue or other discomforts during exercise intervention.

One physical therapist with clinical experience in treating stroke patients for more than five years applied to the tape. The LPTT+PPTT and PPTT groups attached PPTT taping to correct anterior pelvic tilt, and the LPTT+PPTT group was additionally administered LPTT to correct lateral pelvic tilt. In PPTT, I-type strips were attached with 50% tension to activate the EO and RA involved in the posterior pelvic tilt. For mechanical correction, I-type strips (kinesiology 3NS tape, Golden Health Farm, Korea) were attached from the ASIS to the PSIS with a tension of 75%. First, it was attached to both EO muscles from the inguinal region to the spinous process of the 12th thoracic vertebra in the side-lying position and, second, from the ASIS to the PSIS. Finally, in the hook-lying position, it was attached to both RA muscles from the pubic symphysis to the xiphoid process [25]. To apply LPTT, we referred to Maguire's method. For mechanical correction, three I-type strips were attached with 70% tension from just below the greater trochanter to the gluteal surface of the iliac crest in the upward, upward, and anterior, upward, and posterior directions, respectively [19].

### 2.5. Outcome Measure

The anterior and lateral pelvic tilt angles were measured using a palpation meter (PALM). The PALM consists of two caliper arms and is reported to be highly reliable in measuring the height difference between landmarks [26]. In this study, patients stood upright, and their thighs were in contact with a fixed table and then measured. The caliper tip of the PALM was placed on the paralyzed ASIS and PSIS to measure the anterior pelvic tilt angle and on both sides of the ASIS to measure the lateral pelvic tilt angle.

A handheld dynamometer (Model 01163; Lafayette Inc., IN, USA) measured hip abductor strength. We then placed this on the distal third of the most affected femur, and patients were instructed to abduct the hip against it and hold it for 5 seconds. After three measurements, the average value was used for the analysis. The handheld dynamometer has been reported to have high intra- and interrater reliabilities in patients with neurological impairment [27].

A 10-meter walk test (10MWT) was used to measure walking time and speed to complete 10 meters. This measuring tool has been proven to have high intra- and interrater reliability [28].

### 2.6. Data Analysis

Statistical analysis was performed using SPSS (version 21.0; IBM, Armonk, NY, USA), and the normality test of the variables was performed using the Shapiro-Wilk test. A paired *t*-test was performed to confirm intragroup changes, and one-way ANOVA was performed to confirm the difference in the amount of before-and-after change between the three groups. Post hoc analysis was performed using the Bonferroni test. The statistical significance level was set at *p* < 0.05 with 95% confidence limits.

## 3. Results

There was no significant difference among the three groups in terms of general characteristics (Table 1).

**Figure 2:**
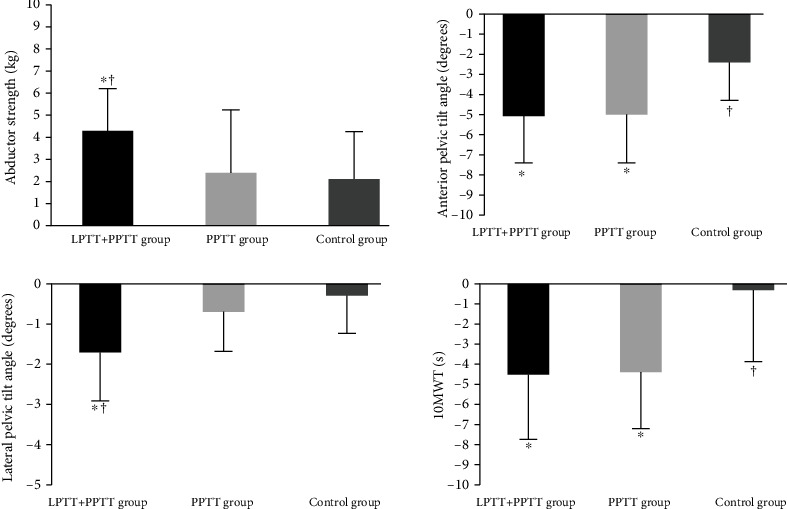
Change values of muscle strength, pelvic inclination, and gait speed in participants. LPTT+PPTT group: lateral pelvic tilt taping and posterior pelvic tilt taping; PPTT group: posterior pelvic tilt; 10MWT: 10-meter walk test. ^∗^Significant difference compared with control group (*p* < 0.05). ^†^Significant difference compared with PPTT group (*p* < 0.05).

After training, abductor strength in the LPTT+PPTT group (change values, 4.3 ± 1.9 kg) showed significantly greater improvement in comparison to that of the PPTT group (change values, 2.4 ± 2.7 kg) and the control group (change values, 2.1 ± 2.0 kg) (*p* = 0.01), and no significant difference between the PPTT and control groups (*p* > 0.05) (Table 2, Figure 2).

The anterior pelvic tilt was significantly improved in the LPTT+PPTT (change values, −4.9 ± 2.2 degrees) and PPTT groups (change values, −4.8 ± 2.4 degrees) in which taping was applied compared to the control group (change values, −2.3 ± 1.8 degrees) (*p* < 0.001), and the lateral pelvic tilt was significantly improved in the LPTT+PPTT group (change values, −1.8 ± 1.4 degrees) compared to the LPTT+PPTT (change values, −0.7 ± 1.0 degrees) and PPTT groups (change values, −0.3 ± 0.9 degrees) (*p* < 0.001) (Table 2, Figure 2).

The gait speed was significantly improved in the LPTT+PPTT (change values, −4.7 ± 3.4 seconds) and PPTT groups (change values, −4.6 ± 2.9 seconds) compared to the control group (change values, −1.8 ± 4.2 seconds) (*p* = 0.02), and there was no significant difference between the LPTT+PPTT and PPTT groups (*p* > 0.05) (Table 2, Figure 2).

## 4. Discussion

This study investigated the parallel effects of PSE and pelvic orthodontic taping. As a result, hip abductor strength was significantly improved in all three groups, and the LPTT+PPTT group showed stronger muscle strength than the other two groups. Dubey et al. reported that hip muscle strength improved after pelvic stability training, which was related to the reorganization of muscle fiber structure as an adaptive response to postural alignment and recruitment of more motor units [23]. This study confirmed that this treatment effect was further strengthened by applying taping for pelvic correction along with PSE, which increases the possibility of cross-bridges by stretching the muscle belly and increasing the overlap of actin and myosin filaments. Therefore, a stronger contraction force can be generated as the length-tension curve is shifted to the left [29]. Another possibility is an increase in muscle activity due to stimulation of cutaneous receptors [30]. Although there was no statistically significant difference between the PPTT and control groups in this study, the hip abductor muscle strength increased more in the two groups to which the taping was applied than in the control group. This result is presumed to be due to the application of PSE, which is helpful for pelvic support and correction, even in the control group. Darak et al. reported that an increase in anterior pelvic tilt on the most affected side was associated with poor control of the lower trunk and weakness of the hip extensors and abductors [31]. In addition, the LPTT+PPTT group tape was directly attached to the abductor muscle, an additional tactile stimulation was provided, and this effect was improved.

Sufficient stabilization of the spine for static and dynamic normal posture is achieved by passive osteoligamentous, active musculotendinous, and neural control systems [32]. In patients with stroke, the alignment of the posterior two systems is altered, and the postural symmetry is disturbed [33]. In this study, as a result of measuring the change in pelvic alignment after training, the anterior pelvic tilt was significantly improved in the LPTT+PPTT group and the PPTT group compared to the control group, and the lateral pelvic tilt was significantly improved in the LPTT+PPTT group compared to the other two groups. The reason that the pelvic alignment of the two groups to which taping was applied in this study improved more is thought to be because the abdominal or hip muscles involved in pelvic tilt were strengthened by applying corrective pelvic taping. Although abdominal muscle or hip extensor strength was not measured in this study, the hip abductor strength of the LPTT+PPTT group was significantly higher than that of the other two groups, and it is thought that this affected the improvement of lateral pelvic tilt. In a study examining the effect of taping on the abdominal muscles in patients with low back pain with increased lordosis, it was also reported that the anterior pelvic tilt decreased as the activation of RA and EO increased [34]. In addition, Levine and Whittle studied the change of the anterior tilt angle according to the measurement posture. As a result, the anterior pelvic inclination angle increased by 11.4 degrees in the maximum anterior tilting posture compared to the normal standing posture. It decreased by 8.7 degrees in the maximum anterior tilting posture. Therefore, they argued that the change in the anterior pelvic tilt angle according to posture supports the fact that pelvic exercise significantly affects lumbar lordosis [35]. Another reason for the improved pelvic alignment is thought to be enhanced proprioception. Unlike the traditional method, the taping method applied to the pelvis has an elasticity exceeding the original length [36], and this elasticity increases overall joint movement, skin deformation [37], and stimulation of cutaneous mechanoreceptors [38]. Because PPTT attaches an I-type strip with a tension of 75% from ASIS to PSIS in the posterior pelvic tilt position, whenever the pelvis is tilted anteriorly, the tape is stretched to generate resistance to the anterior pelvic tilt [34]. This sensory input continuously provides feedback on correct posture and improves proprioception, so it is thought that it may have affected the alignment of the pelvis in the standing posture. However, in the case of anterior pelvic tilt in this study, the two groups with taping were significantly improved compared to the control group, but there was no significant difference between the LPTT+PPTT and PPTT groups. Both groups applied PPTT, and it is considered that the additional application of LPTT is not more effective in reducing anterior tilt. Several studies reported that patients with stroke had an increased pelvic tilt angle compared to healthy participants [39, 40], but there was a considerable variation between studies. Preece et al. suggested that pelvic tilt is determined by the balance of muscular and ligamentous forces acting between the pelvic and adjacent segments, but variation in pelvic morphology may also have a significant effect on the measurement of pelvic tilt [41]. In a study of healthy adults measuring the pelvic angle with a palpation meter, 85% of males and 75% of females showed an anterior pelvic tilt (mean 6–7 degrees), and the rest showed a posterior pelvic tilt or a neutral posture. Therefore, a single pelvic tilt angle could not be the sole cause of the pathology, and the MDC was reported to be 2.5 degrees [42]. Based on these studies' results, it is difficult to define the normal range for a pelvic tilt, and it is thought that it should be evaluated in relation to functional ability. In this study, the amount of change in the anterior pelvic tilt angle of the two groups to which taping was applied and the control group showed a difference of more than 2.5 degrees, which is a clinically meaningful change.

The lateral pelvic tilt is pelvic rotation about the anteroposterior axis, indicating movement in the coronal plane [43]. This occurs when weight is placed on one lower extremity, and this movement is controlled by the hip abductor [44]. During gait in patients with stroke, excessive pelvic motion appeared due to hip muscle weakness [45]. Kim and Eng reported that hip abductor strengthening exercise improved stability in the frontal plane and increased gait speed [46]. In this study, the change in walking speed after training was measured, and it was confirmed that the LPTT+PPTT and PPTT groups were significantly improved compared to the control group. This is thought to be because the increase in hip abductor strength and the efficient improvement of pelvic control during gait reduced unnecessary energy consumption during gait. Lennon reported that pelvic reeducation improved knee extension during the stance phase and ankle motion during the swing phase by reducing anterior pelvic tilt [47]. Studies to improve gait in stroke patients mainly include lower extremity strengthening, electrotherapy, and gait training on a treadmill or in water. Saleh et al. reported that aquatic exercise significantly improved balance, gait, and step length more than dry land training. [48]. And in a study that applied functional electrical stimulation (FES) to gait, it was reported that FES increased gait symmetry by increasing muscle activity suitable for the gait cycle [49]. However, in this study, there was no significant difference in gait speed between the LPTT+PPTT group and the PPTT group after training. This result is thought to be because the difference in the hip abductor muscle strength between the two groups was not large enough to affect the walking speed. Furthermore, there was no significant difference in walking speed between the groups due to other factors, such as muscle strength, ankle movement control, and proprioception.

This study confirmed that muscle activation, pelvic alignment, and gait speed were significantly improved after training by applying LPTT and PPTT with PSE compared to PPTT alone. However, it is difficult to generalize the study results due to the small number of patients. There may be a bias in interpreting the results because there is no natural history group. In addition, it was not confirmed whether weight distribution or gait symmetry improved after training. In future studies, it will be necessary to analyze the different Spatiotemporal variables of various gaits, including the single limb support phase after applying pelvic tilt taping and the effect on the angle of the pelvis or lower extremity joint during gait.

## 5. Conclusion

Our study demonstrated that taping the pelvis improves pelvic alignment. When LPTT and PPTT are applied together, it is more effective in strengthening the abductor muscles and pelvic alignment in the frontal plane. PSE is a training method that can improve pelvic stabilization without requiring special equipment. In clinical practice, combining PSE with taping is a cost-effective treatment for improving pelvic alignment.

## Figures and Tables

**Table 1:** Common and clinical characteristics of participants.

Variable	LPTT+PPTT group (*n* = 20)	PPTT group (*n* = 20)	Control group (*n* = 20)	*p* value
Sex (male/female)	11/9	13/7	11/9	0.760
Age (years)	51.1 ± 8.9^a^	52.2 ± 9.0	52.2 ± 9.0	0.926
Weight (kg)	66.1 ± 8.3	65.9 ± 8.9	64.00 ± 8.8	0.700
Height (cm)	165.4 ± 8.5	165.9 ± 9.1	167.2 ± 9.4	0.812
Stroke type (infarction/hemorrhage)	9/11	14/6	11/9	0.275
Affected side (right/left)	10/10	12/8	9/11	0.627
Poststroke duration (month)	8.8 ± 2.7	9.5 ± 2.0	9.2 ± 2.6	0.656

LPTT+PPTT group: lateral pelvic tilt taping and posterior pelvic tilt taping; PPTT group: posterior pelvic tilt. ^a^Values are expressed as the mean ± standard deviation (SD).

**Table 2:** Participant scores before and after intervention.

Variables	LPTT+PPTT group (*n* = 20)			PPTT group (*n* = 20)			Control group (*n* = 20)			*p*
	Pretest	Posttest	ES	Pretest	Posttest	ES	Pretest	Posttest	ES	
Abductor strength (kg)	9.6 ± 2.7^a^	13.8 ± 3.0	1.47	10.2 ± 3.3	12.6 ± 2.2	0.82	9.8 ± 3.3	11.9 ± 2.2	0.72	0.010
Anterior pelvic tilt (°)	12.6 ± 4.5	7.7 ± 3.1	1.22	11.3 ± 3.9	6.4 ± 2.6	1.42	11.5 ± 4.3	9.2 ± 3.2	0.59	<0.001
Lateral pelvic tilt (°)	5.2 ± 2.6	3.4 ± 1.8	0.78	4.8 ± 2.8	4.1 ± 2.6	0.26	5.0 ± 3.0	4.7 ± 2.5	0.11	<0.001
10MWT (seconds)	26.2 ± 6.1	21.5 ± 4.4	0.86	24.8 ± 4.8	20.2 ± 4.1	1.02	25.4 ± 6.2	23.6 ± 4.8	0.32	0.020

LPTT+PPTT group: lateral pelvic tilt taping and posterior pelvic tilt taping; PPTT group: posterior pelvic tilt; 10MWT: 10-meter walk test; ES: Effect size. ^a^Values are expressed as the mean ± standard deviation (SD).

## Data Availability

The data of this study are available from the corresponding authors upon reasonable request.
